# Diagnostic and prognostic value of ^99m^Tc-pyrophosphate SPECT/CT in patients with transthyretin amyloid cardiomyopathy

**DOI:** 10.1186/s41824-026-00301-y

**Published:** 2026-05-15

**Authors:** Hidenobu Hashimoto, Rine Nakanishi, Shunsuke Kiuchi, Sunao Mizumura, Yukiko Hashimoto, Takanori Ikeda

**Affiliations:** 1 Department of Cardiovascular Medicine, Toho University Graduate School of Medicine, Toho University Omori Medical Center, 6-11-1, Omorinishi, Ota- ward, Tokyo, 143-8541 Japan; 2 Department of Radiology, Toho University Faculty of Medicine, Tokyo, Japan; 3 Department of Cardiovascular Medicine, Department of Internal Medicine, Faculty of Medicine, Toho University, Toho University Omori Medical Center, Tokyo, Japan

**Keywords:** ATTR-CM, ^99m^Tc-PYP, SPECT/CT, Diagnosis, Prognosis

## Abstract

**Background:**

This study evaluated the diagnostic and prognostic values of Technetium- 99m pyrophosphate (^99m^Tc-PYP) scintigraphy using single-photon emission computed tomography/ computed tomography (SPECT/CT) in patients with transthyretin amyloid cardiomyopathy (ATTR-CM).

**Methods:**

Among 206 consecutive patients with suspected cardiac amyloidosis who underwent ^99m^Tc-PYP scintigraphy using SPECT/CT and had no evidence of monoclonal protein, 20 patients were diagnosed with ATTR-CM based on SPECT/CT interpretation, whereas183 had no diagnostic findings on SPECT/CT interpretation. We calculated ^99m^Tc-PYP activity in the volume of interest (VOI) for the myocardium and upper aorta for the blood pool as a reference. The heart-to-blood ratio (HBR) was calculated by dividing the mean activity in the VOI of the myocardium by that in the VOI of the upper aorta. Receiver-operating-characteristic (ROC) analysis was used to assess the diagnostic value of the Perugini grade, HBR, and heart-to-contralateral ratio (H/CL). A multivariate Cox model was used to assess whether the parameters, including HBR, were associated with all-cause mortality, cardiac death, and hospitalization for heart failure.

**Results:**

By ROC analysis, HBR had the most diagnostic value (HBR area under the curve [AUC]: 0.997, 95% confidence interval [CI] 0.990–1.000 vs. H/CL AUC: 0.9617, 95% CI 0.942–0.981 vs. Perugini grade AUC: 0.918, 95% CI 0.891–0.945). During a mean follow-up period of 531 days, 38 major adverse cardiac events occurred. Multivariable Cox models showed that HBR was the most significant prognostic factor (hazard ratio 2.715, 95% CI 1.185–6.221, *p* = 0.018).

**Conclusions:**

This study demonstrated that HBR calculated by ^99m^Tc-PYP scintigraphy using SPECT/CT could have potential diagnostic and prognostic value in patients with ATTR-CM.

## Background

Transthyretin amyloid cardiomyopathy (ATTR-CM) is a condition in which amyloid fibrils are deposited in the heart, resulting in morphological and functional abnormalities (Dungu et al. 2012). ATTR-CM is subtyped according to the sequence of the transthyretin protein as wild type or hereditary (Maurer et al. 2019). Both subtypes of ATTR-CM have been reported to be associated with poor prognosis, with a median survival of approximately 3.5 years following definitive diagnosis (Connors et al. 2016, Grogan et al. 2016, Mariani et al. 2015). The advent of novel therapies has reduced mortality in patients with ATTR-CM (Maurer et al. 2018). Tafamidis was the first transthyretin tetramer stabilizer approved for the treatment of ATTR-CM (McDonagh et al. 2021). Therefore, it is necessary to develop methods that enable accurate diagnosis and risk stratification.

According to expert consensus recommendations, the diagnosis of ATTR-CM can be confirmed with a positive endomyocardial biopsy or a combination of diagnostic findings, including a positive extracardiac biopsy, technetium-99m pyrophosphate (^99m^Tc-PYP) / ^99m^Tc-3,3-diphoshono-1,2-propanodicarboxylic acid / ^99m^Tc-hydroxymethylenediphoshonate scintigraphy, myocardial uptake of positron emission tomography (PET) amyloid tracers, and echocardiographic and cardiac magnetic resonance (CMR) findings. Alternatively, ATTR-CM can be diagnosed based on positive scintigraphy findings with echocardiographic or CMR findings in the absence of a clonal plasma cell process (Dorbala et al. 2019).

Recently, cardiac uptake on bone scintigraphy with the tracer technetium ^99m^Tc-PYP has been confirmed to have high diagnostic sensitivity and specificity for ATTR-CM (Bokhari et al. 2013, Gillmore et al. 2016). The Perugini grade and the heart-to-contralateral (H/CL) ratio are conventional methods for the non-invasive diagnosis of ATTR-CM (Perugini et al. 2005, Masri et al. 2020). However, standardized acquisition method and interpretation protocols are required to ensure reproducibility across institutions (Dorbala et al. 2019).

The use of single-photon emission computed tomography (SPECT) / computed tomography (CT) has been shown to improve diagnostic performance compared with conventional methods using planar image (Ikoma et al. 2023). However, the diagnostic accuracy and prognostic significance of precise myocardial tracer quantification remain unclear. This study investigated the diagnostic and prognostic value of ^99m^Tc-PYP cardiac scintigraphy using SPECT/CT in patients with ATTR-CM.

## Methods

### Patient population

**Fig. 1:**
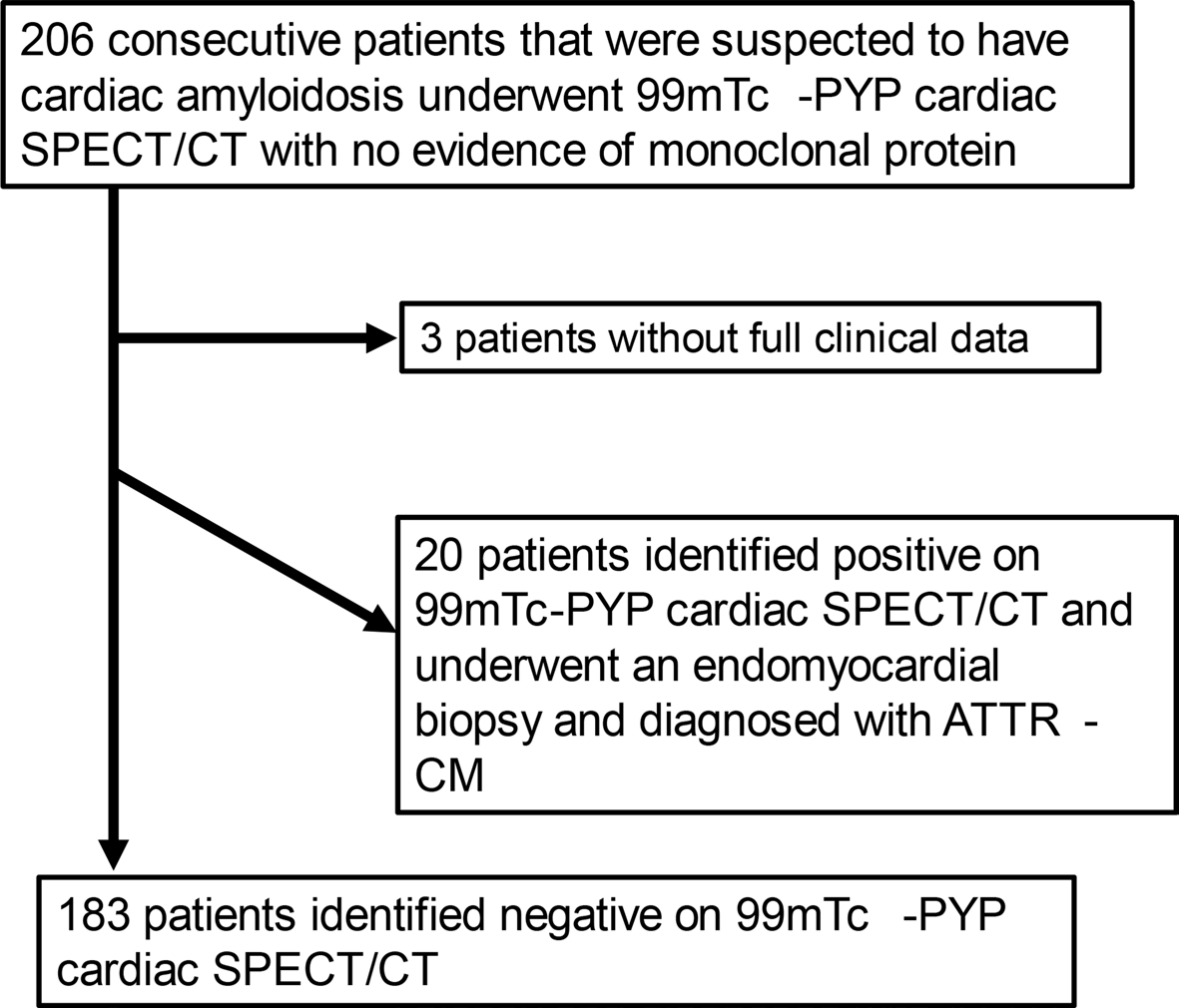
Flow chart of patient inclusion and exclusion criteria. ^99m^Tc-PYP = technetium 99m pyrophosphate; SPECT = single photon emission computed tomography; CT = computed tomography; ATTR-CM = transthyretin amyloid cardiomyopathy

Overall, 206 consecutive patients with suspected ATTR-CM who underwent ^99m^Tc-PYP scintigraphy using SPECT/CT from June 1, 2020, to May 30, 2024, and had at least one of the following tests performed-serum free light chains with its ratio, serum immunofixation electrophoresis, or urinary immunofixation electrophoresis-with no evidence of monoclonal protein were included. Three patients were excluded due to incomplete clinical data. Of 203 patients, 20 patients with positive SPECT/CT findings underwent endomyocardial biopsy, and were diagnosed with ATTR-CM according to the guidelines (Kitaoka et al. 2020). 183 patients had negative SPECT/CT results (Fig. 1). We assessed the patients’ clinical characteristics including age, sex, medical history, blood biochemistry data, echocardiography data, and medications. The Institutional Review Board approved this retrospective study and waived the requirement for informed consent (M24155, 24090).

### Echocardiographic imaging

Echocardiographic images were obtained from the apical window to evaluate the left ventricular function (Vivid E9device, GE Vingmed, Horten, Norway). A thicker interventricular septum (IVS) or left ventricular posterior wall (LVPW) was also measured. Left ventricular ejection fraction (LVEF) was calculated using the Teicholz formula (Lang et al. 2015).

### ^99 m^ Tc-PYP scintigraphy

Thorax planar scintigraphy and SPECT/CT (SPECT with a low-dose, non-contrast CT scan) were performed using a hybrid system (Symbia Intevo, Siemens Healthineers AG, Erlangen, Germany) with low-energy, high-resolusion collimator. The patients received 555 MBq (20 mCi) of ^99m^Tc-PYP intravenously, and images were acquired after 3 h. Thoracic planar images were acquired using a 512 × 512 matrix with a 1.0 zoom factor. Thoracic SPECT/CT images were obtained using a 128 × 128 matrix with a zoom factor of 1.0, 36 projections, and 25 s/projection. After SPECT acquisition, a low-dose CT scan was acquired for attenuation correction (130 kV, 90mAs, 256 × 256 matrix, step-and-shoot acquisition with body contour). Image acquisition and reconstruction were performed using xSPECT/CT QUANT technology (eight iterations, four subsets, a 3.0 mm smoothing filter, and a 20 mm Gaussian filter). The injected dose, residual dose, height, weight, and timing data were entered, and the recorded counts per voxel were converted into activity per unit volume and displayed as an SUV.

### Perugini grade

The intensity of myocardial uptake on planar and SPECT ^99m^Tc-PYP scintigraphy was categorized as 0–3 according to the Perugini grade (Perugini et al. 2005, Dorbala et al. 2019). Grade 0 indicates no uptake or normal bone uptake; grade 1 indicates uptake, which is less intense than the bone signal; grade 2 indicates cardiac uptake with intensity similar to the bone signal; and grade 3 indicates cardiac uptake with intensity greater than the bone signal. Perugini grade ≥ 2 was considered positive.

### H/CL ratio

The H/CL ratio was calculated using the mean count of the circular region of interest (ROI). Planar thoracic image was used to calculate H/CL ratio. ROIs were drawn in the following manner: the region was drawn over the heart, and the contralateral region was drawn in the contralateral chest with an identical size according to ASNC guidelines (Masri et al. 2020). H/CL ratio ≥ 1.3 was considered positive.

### Quantitation of myocardial SPECT/CT images using SYNAPSE VINCENT software

**Fig. 2:**
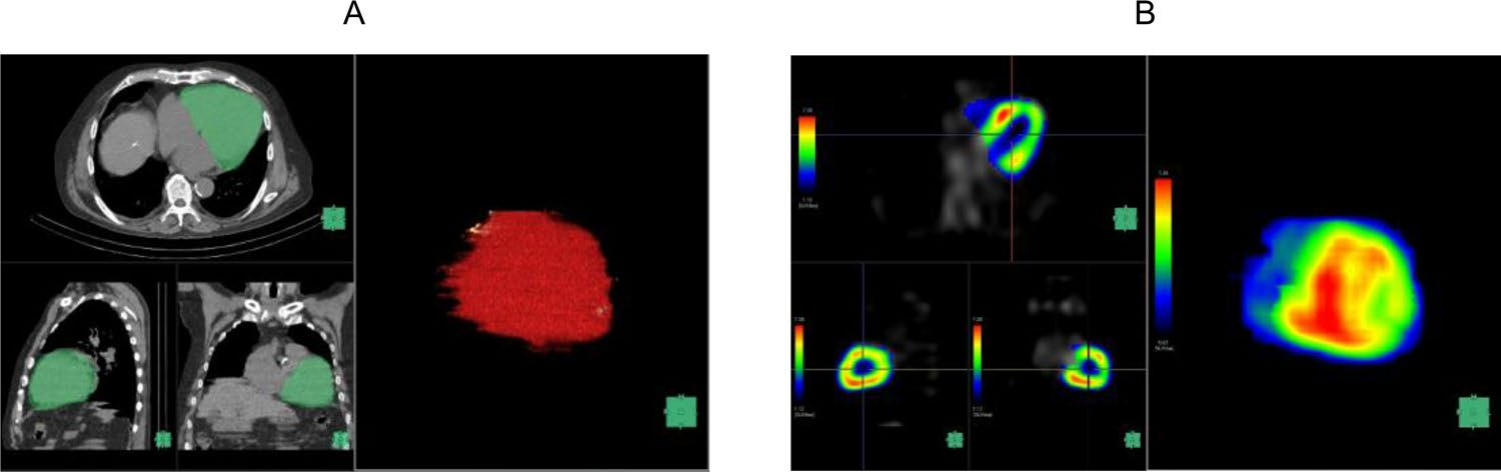
**A** 3D VOI of the heart created by the workstation **B**^99m^Tc-PYP accumulation in the 3D VOI of the image A

**Fig. 3:**
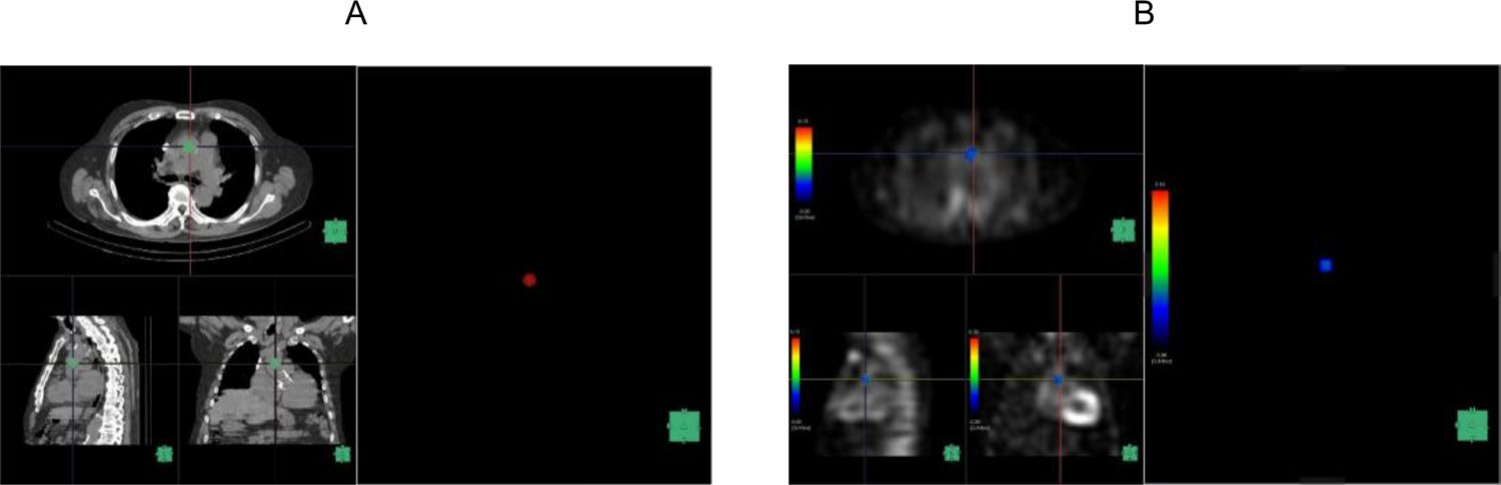
**A** A spherical VOI in the ascending aorta **B**^99m^Tc-PYP accumulation in the image A

We created a volume of interest (VOI) encompassing the combined regions of the left and right ventricular myocardium and cavity using a workstation (SYNAPSE VINCENT; FUJIFILM Medical Co., Ltd, Tokyo, Japan) (Fig. 2A). We then created an image of ^99m^Tc-PYP SPECT/CT in the VOI (Fig. 2B) and a spherical VOI with a diameter of 10 mm, placing it at the level of the pulmonary artery bifurcation within the ascending aorta to measure ^99m^Tc-PYP mean SUV as a reference (Fig. 3A, B). The maximum SUV and mean SUV/VOI values of the myocardium were calculated using voxels within the myocardium with SUV/VOI values greater than the mean SUV/VOI of the aortic VOI. We calculated the heart-to-blood ratio (HBR) by dividing the mean myocardial SUV/VOI by the mean SUV/VOI of the aorta. In the current study, the original reader repeated the measurements in 40 randomly selected patients to evaluate intraobserver reproducibility. Additionally, measurements were repeated in 40 randomly selected patients by another independent reader to assess interobserver reproducibility. The ^99m^Tc-PYP scintigraphy data were interpreted by radiologists board-certified in nuclear medicine. The interpretation of the study and quantitative analysis were performed independently. Neither the patient information nor the other readers’ results were accessible to any of the readers.

### Assessment of clinical outcome

The endpoint was defined as occurrence of major adverse cardiac event (MACE), including cardiac death, which was defined as death caused by heart failure, acute myocardial infarction, lethal ventricular arrhythmias, other definitive cardiac disorders, cardiovascular events (acute myocardial infarction or unstable angina), or severe heart failure requiring hospitalization. Event data were retrospectively collected from patients’ records, including in-hospital or out-of-hospital reports.

### Statistical analysis

Receiver-operating-characteristic (ROC) analysis was used to assess the diagnostic values and incremental diagnostic values of the Perugini grade, H/CL ratio, and HBR. Significance in difference between ROC curves were calculated using Delong test. Data are expressed as average ± standard deviation of continuous variables. Continuous variables from patients with and without events were compared using the Mann–Whitney U test, and categorical data were analyzed using the chi-square test. Age, sex, and factors with a significance level of *P-*value < 0.05 were included in a univariate Cox regression model. Thereafter, variables with significant probable values were included in multivariate Cox regression models to determine whether the future occurrence of MACE was associated with clinical data, parameters of echocardiographic imaging and ^99m^Tc-PYP scintigraphy. Assessment of inter-observer and intra-observer reproducibility was performed using Pearson’s correlation and Bland-Altman analysis. Statistical significance was set at *P* < 0.05, All statistical analyses were performed using STATA for Windows (version 17.0; StataCorp LLC, College Station, Texas, USA).

## Results

The patients’ characteristics, including coronary risk factors, laboratory data, and echocardiographic imaging data are presented in Table 1. The mean age of the 203 patients was 65 ± 15 years, and 150 (74%) were male. Hypertension was the most common comorbidity (*n* = 135, 67%). Table 2 shows comparison of scintigraphy parameters between patients with and without ATTR-CM.

**Table 1:** Patients characteristics

	Total *N* = 203 (%)	Amyloidosis (+) *N* = 20 (%)	Amyloidosis (-) *N* = 183 (%)	*P* value
Age (years)	65.0 ± 15.4	79.8 ± 7.0	63.4 ± 15.3	< 0.001
Male	150 (74)	14 (70)	136 (74)	0.676
Obesity (BMI ≥ 25 kg/m^2^)	74 (36)	7 (35)	67 (37)	0.887
BMI (kg/m^2^)	23.9 ± 5.5	22.8 ± 4.5	24.0 ± 5.6	0.473
Diabetes mellitus	65 (32)	5 (25)	60 (33)	0.479
Hypertension	135 (67)	13 (64)	122 (67)	0.881
Dyslipidemia	54 (27)	9 (45)	45 (25)	0.052
Current smoking	99 (49)	5 (25)	94 (51)	0.025
CKD (eGFR < 60mL/min/1.73m^2^)	91 (45)	13 (65)	78 (43)	0.056
NYHA I/II/III/IV	111/91/1/0	11/9/0/0	100/82/1/0	0.051
AF	47 (23)	7 (35)	40 (22)	0.186
EF	59.4 ± 16.3	49.5 ± 18.4	60.5 ± 15.7	0.009
IVS or LVPW	13.7 ± 3.9	17.2 ± 3.2	13.4 ± 3.8	< 0.001
BNP	435.8 ± 904.1	673.9 ± 787.2	409.7 ± 914.1	0.002
**Medication**				
β	111 (55)	9 (45)	102 (56)	0.360
Diuretics	46 (23)	8 (40)	38 (21)	0.051
CCB	91 (45)	6 (30)	85 (46)	0.160
ACE-I, ARB	126 (62)	9 (45)	117 (64)	0.098
MRA	51 (25)	8 (40)	43 (23)	0.106
SGLT2 inhibitor	54 (27)	7 (35)	47 (26)	0.379

AF = atrial fibrillation; ARB = angiotensin receptor blocker; BNP = brain natriuretic peptide; BMI = body mass index; CCB = calcium channel blocker; CKD = chronic kidney disease; EF = ejection fraction; eGFR = estimated glomerular filtration rate; IVS = interventricular septum thickness; LVPW = left ventricular posterior wall thickness; MRA = mineralocorticoid receptor antagonist; SGLT2 inhibitor = sodium-glucose cotransporters

**Table 2:** Comparison of scintigraphy parameters between patients with and without ATTR-CM

	Total *N* = 203	Amyloidosis (+) *N* = 20	Amyloidosis (-) *N* = 183	*P* value
H/CL ratio	1.22 ± 0.27	1.90 ± 0.23	1.15 ± 0.14	< 0.001
Perugini grade (planar)	1.08 ± 0.93	3.0 ± 0.0	0.87 ± 0.71	< 0.001
Perugini grade (SPECT)	0.92 ± 0.94	3.0 ± 0.0	0.69 ± 0.68	< 0.001
Myocardial max SUV	2.77 ± 1.09	5.09 ± 1.41	2.52 ± 0.69	< 0.001
HBR	1.17 ± 0.25	1.83 ± 0.32	1.10 ± 0.08	< 0.001

HBR = heart-to-blood ratio; H/CL = heart-to-contralateral; SPECT = single-photon emission computed tomography; SUV = standardized uptake value

**Fig. 4:**
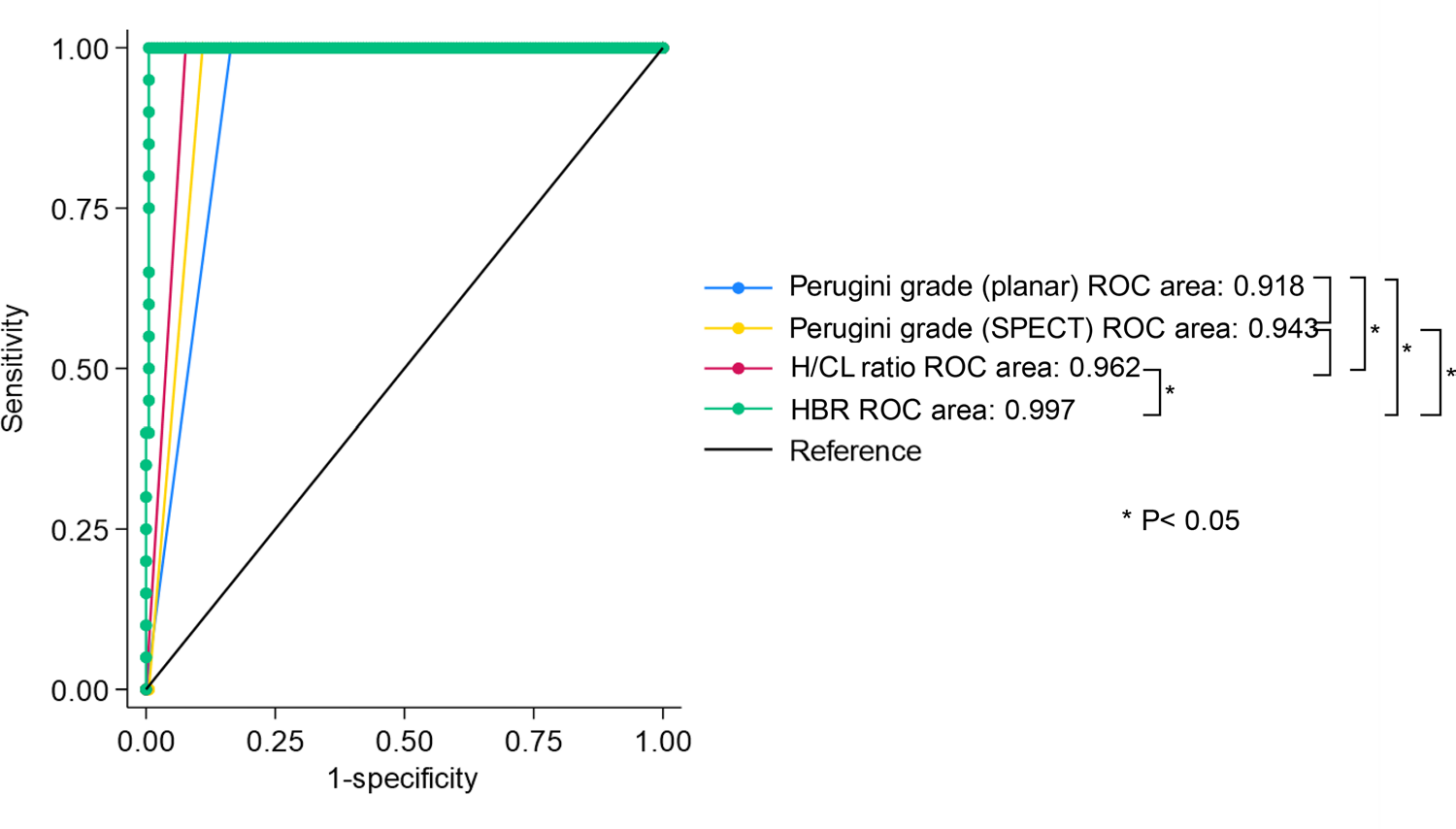
ROC analysis for comparing the AUCs of the measures. H/CL = heart-to-contralateral; HBR = heart-to-blood ratio

**Fig. 5:**
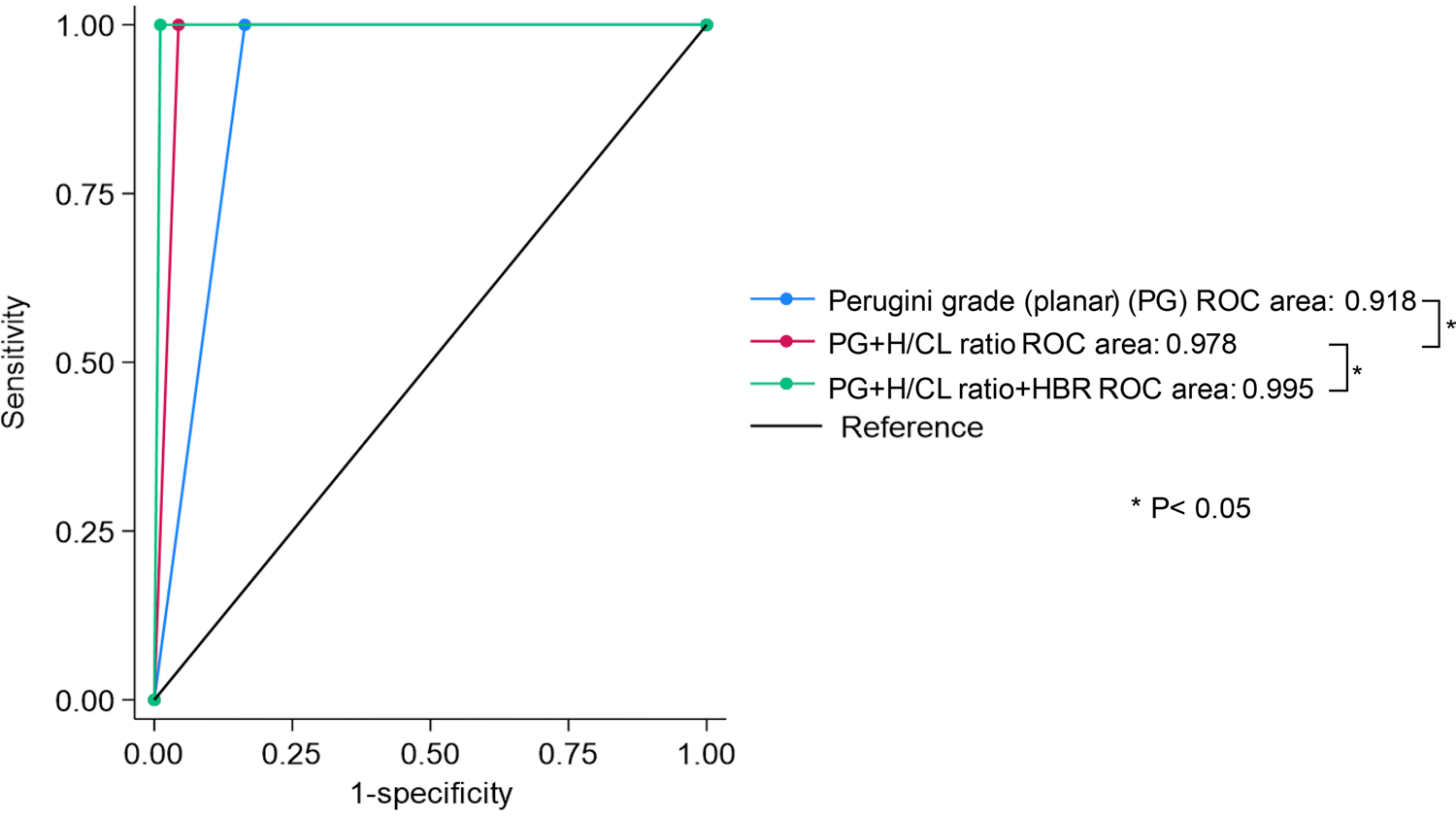
ROC analysis for incremental value of HBR. H/CL = heart-to-contralateral; HBR = heart-to-blood ratio

Based on the ROC analysis, HBR demonstrated the highest diagnostic performance compared to that of the Perugini grade and H/CL ratio (HBR AUC: 0.997, 95% CI 0.990–1.000, cutoff 1.335, sensitivity 100% specificity 99.45% vs. H/CL AUC: 0.962, 95% CI 0.942–0.981, sensitivity 100% specificity 92.35%, *P* < 0.001, vs. Perugini grade planar AUC: 0.918, 95% CI 0.891–0.945, sensitivity 100% specificity 83.61%, *P* = 0.002, SPECT AUC: 0.943, 95% CI 0.918–0.967, sensitivity 100% specificity 89.1%, *P* = 0.193) (Fig. 4). The incremental diagnostic performance for ATTR-CM, Perugini grade planar combined H/CL ratio and HBR was demonstrated greater compared to Perugini grade planar combined H/CL ratio, and Perugini grade planar alone (Perugini grade, H/CL ratio and HBR AUC: 0.995, 95% CI 0.987–1.000, HBR cutoff 1.335 vs. Perugini grade combined H/CL ratio AUC: 0.978, 95% CI 0.963–0.993, *P* = 0.013, vs. Perugini grade planar AUC: 0.918, 95% CI 0.891–0.945, *P* < 0.001) (Fig. 5).

During a mean follow-up period of 531 ± 385 days, 38 patients experienced MACE. All-cause mortality occurred in 18 patients, cardiac death in 5, severe heart failure requiring hospitalization in 13, and cardiovascular events in 2. Age, IVS, LVPW, BNP were significantly higher, and current smoking and EF were significantly lower in patients with ATTR-CM (Table 1). Perugini grade, H/CL ratio, myocardial maximum SUV, and HBR were significantly higher in patients with ATTR-CM (Table 2). In the multivariate analysis, HBR was the most significant independent prognostic factor for MACE (*P* = 0.018) (Table 3). The reproducibility of the measurements was excellent (*r* = 0.99, *P* < 0.001) (Table 4).

**Table 3:** Univariate and multivariate Cox regression analysis for occurrence of MACE

	Univariate analysis			Multivariate analysis (adjusted by Age and BNP) model	
	HR (CI)	*P* value		HR (Cl)	*P* value
Age	1.046 (1.019–1.073)	0.001			
Current smoking	1.634 (0.844–3.163)	0.145			
EF	0.984 (0.964–1.003)	0.100			
IVS or LVPW	1.037 (0.963–1.117)	1.037			
BNP	1.000 (1.000–1.001)	0.015			
H/CL ratio	4.693 (2.063–10.674)	< 0.001		2.610 (1.066–6.393)	0.036
Perugini grade (planar)	1.431 (1.027–1.994)	0.034		1.169 (0.833–1.641)	0.366
Perugini grade (SPECT)	1.878 (1.418–2.488)	< 0.001		1.485 (1.062–2.076)	0.021
Myocardial max SUV	1.273 (1.024–1.583)	0.030		1.201 (0.981–1.471)	0.076
HBR	4.280 (1.996–9.175)	< 0.001		2.715 (1.185–6.221)	0.018

CI = confidence interval; HR = hazard ratio; other abbreviations as in Table 1

Multivariate analysis adjusted for age and BNP

**Table 4:** Intraobserver and interobserver reliability

	Myocardial max SUV	HBR
**Intra-observer**		
**All patients (** ***N*** ** = 40)**		
Coefficient	0.98 (*p* < 0.001)	0.99 (*p* < 0.001)
Limit of agreement	-0.61–0.74	-0.04–0.02
**Inter-observer**		
**All patients (** ***N*** ** = 40)**		
Coefficient	0.99 (*p* < 0.001)	0.99 (*p* < 0.001)
Limit of agreement	-0.09–0.05	-0.09–0.05

Abbreviations as in Table 2

### Case presentation

**Fig. 6:**
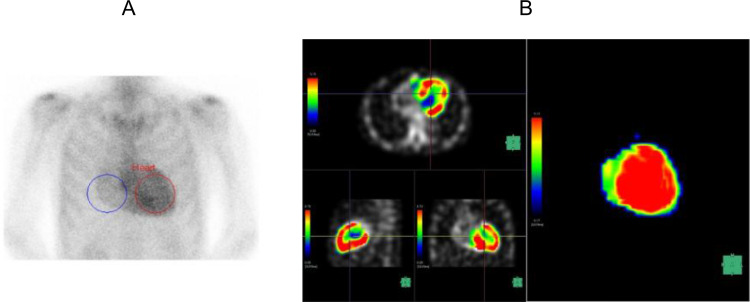
**A** A ^99m^Tc-PYP planar image of a 66-year-old man with ATTR-CM **B**^99m^Tc-PYP accumulation images in 3D VOI created by the workstation of a 66-year-old man with ATTR-CM

Figure 6 (A, B) shows the planar and SPECT/CT images of a 66-year-old man with ATTR-CM. The patient had a history of hypertension and chronic kidney disease. ^99m^Tc-PYP SPECT/CT was performed for suspected cardiac amyloidosis based on the increased wall thickness detected on echocardiography. Both the Perugini grade and H/CL ratio were elevated, and SPECT/CT demonstrated increased tracer uptake in the myocardium. Therefore, the patient underwent endomyocardial biopsy and was diagnosed with ATTR-CM. The HBR was also evaluated with a value of 2.21. In the present case, the patient died of cardiac death owing to deterioration of heart failure after 910 days.

**Fig. 7:**
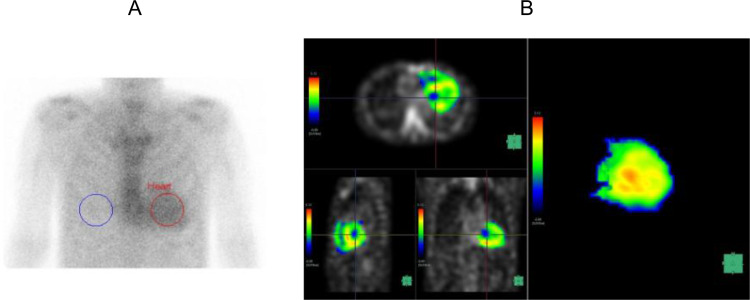
**A** A ^99m^Tc-PYP planar image of a 74-year-old man with ATTR-CM **B**^99m^Tc-PYP accumulation images in 3D VOI created by the workstation of a 74-year-old man with ATTR-CM

Figure 7 (A, B) shows the planar and SPECT/CT images of a 74-year-old man with ATTR-CM. The patient had a history of hypertension, diabetes mellitus and chronic kidney disease. ^99m^Tc-PYP SPECT/CT was performed for suspected cardiac amyloidosis based on the increased wall thickness detected on echocardiography. Both the Perugini grade and H/CL ratio were elevated, and SPECT/CT demonstrated increased tracer uptake in the myocardium. Therefore, the patient underwent endomyocardial biopsy and was diagnosed with ATTR-CM. The HBR was evaluated with a value of 1.33. In the present case, the patient remained event-free for 319 days of follow-up.

## Discussion

To our knowledge, this is the first study to investigate the diagnostic and prognostic values of ^99m^Tc-PYP SPECT/CT in patients with suspected ATTR-CM. Our findings demonstrated that HBR calculated using ^99m^Tc-PYP SPECT/CT could be a potential diagnostic and prognostic marker in patients with suspected ATTR-CM.

### Diagnostic performance of SPECT/CT for ATTR-CM

The Perugini grade has been widely used as a conventional diagnostic method (Miller et al. 2022, Piekarski et al. 2018). Saitou et al. reported that visual score on 3-h had a sensitivity of 80.5% and specificity of 86.8% (Saitou et al. 2023). In this study, the sensitivity and specificity of the Perugini grade were 100% and 83.6%, the differences in these results may have been influenced by the study population. H/CL ratio is a widely used diagnostic method (Bokhari et al. 2013, Pandey et al. 2023). Pandey et al. reported that the sensitivity and specificity of the H/CL ratio were 93% and 99%, respectively (Pandey et al. 2023). Castano et al. reported that the sensitivity and specificity of the H/CL ratio were 91% and 92%, respectively (Castano et al. 2016). In this study, the sensitivity and specificity of the H/CL ratio were 100% and 92.4%, respectively. 97 patients had Perugini grade 1, considered equivocal scan, and all were negative on SPECT/CT interpretation. The HBR values were below the cutoff value of 1.335 in all of these cases. In addition, thirteen patients had an H/CL ratio of 1.3–1.5 and were considered to have equivocal scans, among whom one patient had ATTR-CM. Among these patients, the HBR exceeded the cutoff value only in that patient. A Perugini grade of 2 or 3, or an H/CL ratio greater than 1.3, did not miss any cases of ATTR-CM, but each was associated with false-positive rates of 16.4% and 7.7%, respectively. This can be attributed to the tracer accumulation in the blood pool. Therefore, recent multi-societal guidelines have recommended planar image with SPECT image to confirm myocardial uptake (Dorbala et al. 2019, Dorbala et al. 2019). Asif et al. reported that the sensitivity, specificity, positive predictive value, negative predictive value, and accuracy of ^99m^Tc-PYP SPECT were 97%, 98%, 94%, 99% and 98%, respectively (Asif et al. 2021). Dorbala et al. reported that quantitative metrics derived from ^99m^Tc-PYP using cadmium-zinc-telluride SPECT/CT were strongly correlated with traditional structural markers of cardiac amyloid burden (Dorbala et al. 2021). In this study, we scored Perugini grade using planar and SPECT images, respectively. A potential explanation for the lack of a statistically significant difference in diagnostic performance between the planar and SPECT images is that the SPECT image alone, without the CT image, may not completely exclude tracer uptake within the blood pool. This finding is further supported by the highest diagnostic performance of HBR, with the sensitivity and specificity were 100% and 99.5%, respectively in this study. These findings are consistent with those of previous reports and suggest that quantitative assessment using SPECT/CT improves the diagnostic accuracy (Scully et al. 2020, Ramsay et al. 2018). By excluding activity from the intracardiac blood pool from the total cardiac activity, this study enabled a more precise evaluation of myocardial tracer uptake, which is considered to have contributed to favorable outcomes. Therefore, HBR derived from SPECT/CT may reduce false positive and unnecessary biopsies, thereby mitigating clinical risks in this elderly population.

### Prognostic value of SPECT/CT for ATTR-CM

In this study, an HBR ≥ 1.335 predicted worse survival (hazard ratio, 2.715; *P* = 0.018). Previous studies reported an association between troponin and N-terminal pro-B-type natriuretic peptide levels and prognosis (Grogan et al. 2016, Hutt et al. 2017, Gillmore et al. 2018). However, in the present study, BNP level was significantly associated with prognosis in the univariate Cox analysis, and no significant association was observed in the multivariate analysis. Castano et al. reported that an H/CL ratio of ≥ 1.6 predicted worse survival and in Kaplan-Meier analysis over a 5-year-follow-up period, survival was significantly worse if the H/CL ratio was ≥ 1.6 (Castano et al. 2016). These results do not contradict our data. In the present study, HBR was considered a superior prognostic predictor than H/CL. This may be attributed to the use of CT images to generate 3D VOI in this study, which enabled the evaluation of tracer uptake exclusively in the myocardium after eliminating the influence of the blood pool.

However, the molecular mechanism whereby ^99m^Tc-PYP selectively binds to ATTR amyloid fibrils in the myocardium remains unknown. Several underlying mechanisms are considered. First, ^99m^Tc-PYP binds to amyloid deposits in a calcium-dependent manner (Pepys et al. 1979). Second, in myocardial infarction lesions, ^99m^Tc-PYP is most prominently visualized between 48 and 72 h and becomes undetectable after 1 week (Buja et al. 1977). Therefore, in cardiac amyloidosis, the uptake of ^99m^Tc-PYP may reflect the disease activity at the time of examination, these hypotheses are consistent with our findings. Further studies are needed to confirm whether HBR can help in the diagnosis of ATTR-CM, may obviate the need for biopsy, and offers a means of monitoring the response to therapy in a large patient population.

### Study limitations

This study had some limitations. First, the number of patients with ATTR-CM was relatively small, limiting the statistical reliability of the study. However, our results demonstrate that HBR is a significant diagnostic and prognostic indicator. Second, although the aorta was used as the reference region in this study, further investigations are required to determine the optimal reference region. Previous studies have suggested that tracer uptake in the aorta shows less variability and lower values than in other regions, supporting it as an optimal reference region (Ramsay et al. 2018). Third, we lacked data on troponin levels from laboratory tests and global longitudinal strain calculated using echocardiography. These are the major factors for evaluating ATTR-CM. Fourth, in this study, we could not assess whether the HBR is superior to SPECT/CT interpretation for diagnosis of ATTR-CM. Thus, prospective studies including all patients who underwent endomyocardial biopsy and incorporating the above clinical data before and after treatment are warranted, and are the focus of our ongoing investigations.

## Conclusion

In this study, HBR calculated using ^99m^Tc-PYP SPECT/CT could potentially have diagnostic and prognostic values in patients with ATTR-CM.

## Data Availability

The datasets used and analyzed during the current study are available from the corresponding author on reasonable request.

## Abbreviations

ATTR-CM: Transthyretin amyloid cardiomyopathy
^99m^Tc-PYP: Technetium 99m pyrophosphate
H/CL: Heart-to-contralateral
SPECT/CT: Single-photon emission computed tomography/ computed tomography
LVEF: Left ventricular ejection fraction
ROI: Region of interest
VOI: Volume of interest
HBR: Heart-to-blood ratio
MACE: Major adverse cardiac event
ROC analysis: Receiver-operating-characteristic analysis
